# Recent Advances in Lateral Flow Immunoassay for Rapid Diagnosis of Viral Diseases

**DOI:** 10.1155/tbed/5701806

**Published:** 2026-01-10

**Authors:** Quanyu Ren, Yan Wang, Haoyuan Ma, Jialiang Xie, Jianyou Jin, Rumeng Tian, Hao Yu, Xu Gao

**Affiliations:** ^1^ College of Agriculture, Yanbian University, Yanji, Jilin, 133002, China, ybu.edu.cn

**Keywords:** lateral flow immunoassay (LFIA), on-site detection, point-of-care test (POCT), virus

## Abstract

Viral diseases are a major threat to human and animal health, as illustrated by recent pandemics like COVID‐19 and African swine fever (ASF). Timely, accurate detection of viral infections is critical for effective disease control. Among diverse diagnostic techniques, lateral flow immunoassay (LFIA) has become a widely used on‐site testing tool, owing to its speed, simplicity, affordability, and portability. The application of LFIA for detecting human and animal viruses is feasible, which highlights its practical utility in veterinary settings. This review summarizes key advances in LFIA for the rapid diagnosis of viral diseases over the past decade, focusing on its technical principles, practical applications, core advantages, existing limitations, and potential effective strategies to provide comprehensive knowledge for virus detection.

## 1. Introduction

Viral diseases remain a persistent and evolving threat to global public health, with their impact amplified by factors such as urbanization, international travel, climate change, and the emergence of novel viral strains. Over the past decade, the world has witnessed multiple viral outbreaks, such as the Ebola virus epidemic in West Africa [1], the 2015–2016 Zika virus pandemic [2], and the COVID‐19 pandemic caused by SARS‐CoV‐2 [3, 4]. Beyond these large‐scale outbreaks, endemic viral diseases, such as influenza [5], hepatitis B and C [6], porcine epidemic diarrhea (PED) [7, 8], and African swine fever (ASF) [9, 10], continue to impose a heavy burden on humans or animals, with numerous new infections reported annually [11, 12]. Furthermore, as reported by the World Organisation for Animal Health (WOAH), a total of 31 outbreaks of Avian influenza in poultry, 37 in nonpoultry birds and mammals occurred in Africa, the Americas, Asia, and Europe during September 2025, resulting in more than 907,222 poultry being either killed or culled (https://www.woah.org/en/home/). From January 1, 2022, to August 31, 2025, ASF has been reported as present in four different world regions across 68 countries, affecting 1,079,278 pigs and 39,161 wild boars, resulting in 2,255,137 animal losses. These data indicate that up‐to‐date animal health information and insights into the emergence and progression of viral diseases are particularly critical for the aquaculture and livestock industry.

Timely and accurate diagnosis is crucial for effective viral disease management, which enables the early isolation of infected individuals to prevent transmission, the targeted administration of antiviral treatments, and the rapid implementation of outbreak control measures (such as vaccination and travel restrictions) [13]. Traditional laboratory‐based diagnostic methods, such as reverse transcription‐polymerase chain reaction (RT‐PCR) and real‐time quantitative PCR (qPCR) for nucleic acid detection and enzyme‐linked immunosorbent assay (ELISA) for antigen/antibody detection, are effective methods for virus detection. However, PCR and ELISA require specialized equipment, trained personnel, and long turnaround times (2–24 h), which limits their use in remote clinics/farms, disaster zones, or low‐ and middle‐income countries, especially in resource‐constrained settings or emergency scenarios.

Lateral flow immunoassay (LFIA) has served as a transformative point‐of‐care (POC) diagnostic tool [14–16]. Its advantages, including simplicity, rapidity, cost‐effectiveness, and portability, enable the assay to be widely used for POC test (POCT), particularly in settings with limited healthcare resources. LFIA’s ability to detect viral antigens, antibodies, or even nucleic acids has made it a versatile tool for both acute infection screening and serological surveillance. During the COVID‐19 pandemic, the LFIA proved to be a feasible and effective method for disease surveillance [17, 18] or seroprevalence survey [19]. Aboagye et al. [20] reported that RT‐PCR and antigen rapid diagnostic tests (Ag‐RDTs), such as fluorescence immunoassays (FIAs) and LFIA, exhibit comparable performance in detecting SARS‐CoV‐2. This review, based on literature publicly available through PubMed, discusses the latest research progress in LFIA for the diagnosis of viral diseases over the past decade. We also compare LFIA with alternative diagnostic methods, highlighting its advantages and limitations. Additionally, innovative solutions to overcome drawbacks, such as nanomaterial‐based signal amplification and microfluidic integration, are also proposed, aiming to offer insights into the future role of LFIA in viral disease surveillance and response.

## 2. Technical Principles of LFIA

### 2.1. Basic Components of LFIA

**Figure 1:**
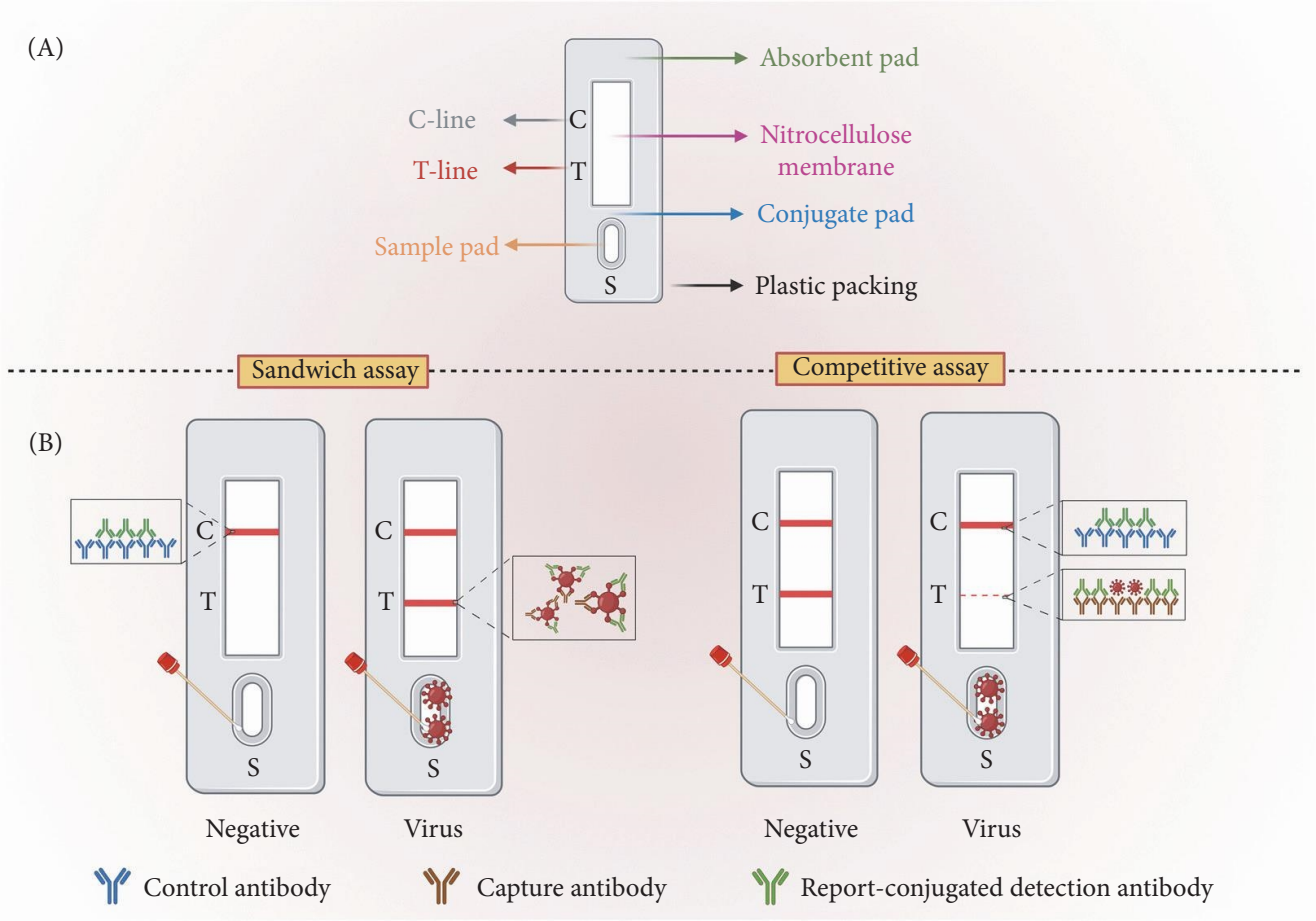
General design and working mechanism of LFIA. (A) Schematic diagram of a typical LFIA strip. The strip contains a sample pad (S), a conjugate pad (containing reporter‐labeled detection antibody), a nitrocellulose (NC) membrane with test (T) and control (C) lines, and an absorbent pad on a plastic backing. (B) Working mechanisms of the LFIA. Sandwich LFIA: The target analyte binds labeled probes on the conjugate pad, forming complexes that are trapped at the T‐line (a sandwich structure with capture probes). Unbound probes bind the C‐line. Positive results show dual T/C‐line signals; negative results show only C‐line. Competitive LFIA: the target analytes in the sample compete with the labeled reporter preloaded on the conjugate pad for binding to the preimmobilized capture agents on the T‐line. A weak or undetectable T‐line indicates a high target concentration in the sample (positive result), while a strong T‐line suggests a low or absent target concentration (negative result).

A typical LFIA strip consists of five key parts, including a sample pad, conjugate pad, nitrocellulose (NC) membrane, absorbent pad, and plastic packing (Figure 1A) [14, 15]. The sample pad serves as the starting point for adding the sample (such as blood, saliva, or nasal swab extract), designed to absorb and spread it evenly across the strip rapidly. The conjugate pad was preloaded with detection probes (including nucleic acids, antibodies, or antigens), which are conjugated with reporter substances, including fluorescent microspheres, quantum dots (QDs), gold nanoparticles (GNPs), or magnetic nanoparticles (MNPs). The NC membrane contains two important lines, the test line (T‐line) and the control line (C‐line). The T‐line is precoated with a capture probe (nucleic acids, antibody, antigen, or ligand) specific to the target analyte or its conjugates. The C‐line is preloaded with a universal binding agent (such as an anti‐IgG antibody) that can interact with the labeled probes regardless of the presence of the target analyte, serving as an internal control to ensure the proper functioning of the assay. The absorbent pad is located at the end of the strip to absorb excess sample and liquid, thereby preventing backflow and ensuring continuous capillary flow through the strip.

### 2.2. Working Mechanisms

There are two main working mechanisms in LFIA: the sandwich and the competitive assays (Figure 1B). For the sandwich assay, upon loading a sample containing the target analyte onto the sample pad, the fluid moves through the conjugate pad, where the target analyte binds to the labeled probes [21, 22]. Subsequently, as the complex migrates along the strip, this analyte–probe complex moves to the T‐line, where it is trapped and assembles into a sandwich structure with the immobilized capture probe. Furthermore, the redundant detection probe continues to move and binds to the universal binding agent on the C‐line. Thus, the labeled complexes accumulated on the C‐line and/or T‐line. Depending on the characteristics of the reporter conjugated to the detection probes, enzyme‐mediated or light‐triggered activation then leads to the formation of a visible signal at the C‐line and/or T‐line. In this circumstance, the accumulation of the labeled complexes on the T‐line indicates the presence of the target virus. The intensity of the signal is typically positively associated with the concentration of the targets in the sample. Notably, the detection and capture probes should be distinct, such as different monoclonal antibodies (mAbs).

In a standard competitive LFIA, the target analytes in the sample compete with the labeled reporter preloaded on the conjugate pad for binding to the preimmobilized capture agents on the T‐line [23]. The labeled reporter can also bind to the universal binding agents on the C‐line. Therefore, in negative cases, the labeled reporters occupy all the binding sites on the capture agents and the universal binding agents, resulting in visible signals at both the T‐line and the C‐line. However, in positive cases, sample targets bind to the capture agents on the T‐line, leaving fewer binding sites available for the reporters, thereby impeding the accumulation of complexes on the T‐line. As a result, the signal intensity on the T‐line is inversely correlated with the concentration of the target in the sample. A weak or undetectable T‐line indicates a high target concentration in the sample (positive result), while a strong T‐line suggests a low or absent target concentration (negative result).

## 3. Applications of LFIA in Viral Disease Diagnosis

In the clinic, LFIA is used to detect viral nucleic acids, antigens, and antibodies. This multiple functionality enables the assay to be used for various stages of viral infection and clinical needs, making it a versatile POCT tool (Table 1).

**Table 1:** Summary of LFIAs developed for specific viruses.

Virus	Design strategy	Assay time	LOD range	Accuracy	Ref.
SARS‐CoV‐2	SERS‐based LFIA. The immune‐SERS tags, 4‐MBA‐modified Au@Ag NPs, are on the conjugate pad. Anti‐N protein antibody and goat anti‐mouse IgG are loaded on the T‐ and C‐line, respectively.	12 min	2.16 pg/mL	Can detect the N or spike proteins within 1–3 days of infection. Consistent with those of RT‐PCR, 150 times more sensitive than conventional colorimetric LFIA.	[24]
	Nanobody‐based LFIA. N protein‐specific nanobody fused with GNPs and IgY antibody‐conjugated GNPs are loaded on the conjugate pad. Fc nanobody for T‐line and IgY for C‐line.	15 min	10^3.15^ PFU/mL	Comparable to that of RT‐qPCR, and 25‐fold higher sensitivity than the commercial antibody‐based LFIA. Stable for 6 weeks at RT.	[25]
	Triple‐antibody sandwich LFIA targeting the viral N or RBD. mAbs and nanobodies were labeled with TRF and streptavidin‐biotin and preloaded on the conjugate pad. Viral N or RBD is on the T‐line, goat anti‐rabbit antibody is on the C‐line.	15 min	12.01 pg/mL for N protein and 6.51 pg/mL for RBD using a portable fluorescence strip reader	4–32‐fold enhancements in sensitivity, and 32–256‐fold enhancements compared to commercial antigen LFIA.	[26]
	Multiplex RT‐LAMP‐LFA assay for differentiating SARS‐CoV‐2 variants, particularly the Omicron BA.1 and BA.2 lineages. Biotin‐5′‐FIP primer for BA.1 LAMP primer, digoxigenin‐5^′^‐FIP prime for BA.2 LAMP primer, and FAM‐5^′^‐LF primers in both LAMP primer sets. The HybriDetect2T lateral flow assay kit (Milenia Biotec GmbH, Germany) was used in this study. HybriDetect2T buffer contains anti‐FAM antibodies‐GNPs. Anti‐rabbit antibody is on the C‐line. The biotin ligand and anti‐Digoxigenin antibody are on the T1 and T2 Lines, respectively.	40 min	1.0 × 10^3^ copies/μL for the BA.1 and 7.5 × 10^2^ copies/μL for BA.2	BA.1: 66%–100% sensitivity, 79%–100% specificity, and 86%–100% accuracy; BA.2: 59%–100% sensitivity, 73%–100% specificity, and 80%–100% accuracy.	[27]
	SERS‐based LFIA for the simultaneous detection of IAV, SARS‐CoV‐2, and respiratory syncytial virus. Dual‐layer DTNB‐modified Fe_3_O_4_@Au MNPs were coupled with the anti‐H1N1/SARS‐CoV‐2/RSV antibodies and incubated with the sample. The mixture is enriched by a magnet and loaded on the strip. The antibodies, goat anti‐mouse IgG, H1N1, SARS‐CoV‐2, and RSV, are sprayed on C‐line, T1, T2, and T3 lines, respectively.	30 min	85 copies/mL, 8 pg/mL, and 8 pg/mL for H1N1, SARS‐CoV‐2, and RSV, respectively.	100% specificity	[28]
PEDV	Primers are labeled with digoxin and FAM, generating digoxin‐ and FAM‐conjugated amplicons after a one‐step RT‐PCR. Amplicon can be captured by the colloidal gold‐anti‐FAM antibody on the conjugated pad and immobilized by the anti‐digoxin antibody, resulting in a colloidal gold‐anti‐FAM antibody‐viral amplicon‐anti‐digoxin antibody structure on the T‐line.	45 min	114.1 copies	100% specificity	[29]
	Multiplexed fluorescence LFIA for differentiating PEDV, PDCoV, and TGEV. The assay is characterized by preloaded europium NPs‐conjugated antibodies specific to PDCoV, TGEV, and PEDV on the conjugated pad, and virus‐specific antibodies on three T‐lines.	15 min	PDCoV, TGEV, and PEDV were 2.1 × 10^4^ TCID_50_/mL, 3.4 × 10^2^ TCID_50_/mL, and 3.6 × 10^2^ TCID_50_/mL, respectively	Coincidence rate to RT‐qPCR: 90%. 100% specificity. Good stability for at least 23 days.	[30]
	Triplex RT‐RAA‐LFIA to differentiate PEDV, PDCoV, and TGEV. Reverse primers targeting the three viruses are labeled with distinct fluorophores (Alexa Fluor 488, digoxigenin, and 6‐carboxyfluorescein), while specific probes bear a biotin label. The amplicons are mixed with streptavidin‐coated colloidal gold. T‐line is immobilized with antibodies corresponding to each fluorophore.	15 min, including 10 min for RAA, 5 min for LFIA.	1 × 10 TCID_50_ PEDV, 1 × 10^4^ TCID_50_ PDCoV, and 1 × 10^2^ TCID_50_ TGEV per reaction, respectively.	100%, 99.1%, and 99.1% consistency with qPCR in clinical testing, respectively.	[8]
	SERS‐LFIA: 4‐MBA and PEDV antibodies were immobilized on the surface of the gold nanoantennas and preloaded on the conjugated pad to bind PEDV virions. Then, the PEDV antibody on the T‐line detects the virus–NPs complex.	20 min	Naked eye or a Raman spectrometer: 1.0 × 10^2^ and 1.0 TCID_50_/mL, respectively	94.91%–102.1% specificity	[31]
Influenza Virus	This system contains freeze‐dried probes and an LFIA strip. Simultaneous differentiation of SARS‐CoV‐2, ADV, and IAV using multiplex fluorescence LFIA. Viral‐specific mAbs conjugated with QDs are freezed probes to incubate with the diluted sample. Then, the mixture was added to the sample pad to interact with the capture antibody on the T‐line. The three T‐lines and one C‐line were precoated with SARS‐CoV‐2, ADV, and IAV capture antibodies, as well as polyclonal goat anti‐mouse IgG, respectively.	20 min	For naked eye evaluation: 160 copies/mL for SARS‐CoV‐2, 1000 copies/mL for ADV, and 200 copies/mL for IAV. For portable reader: 56 copies/mL for SARS‐CoV‐2, 120 copies/mL for ADV, and 41 copies/mL for IAV.	100% specificity. Compared to colloidal gold LFIA, the LOD was improved by 200, 417, and 1220 times, respectively, while maintaining sensitivity comparable to that of PCR. Stable for 8 months.	[32]
	LAMP‐LFIA: After lysis and elution using the squeeze method, place one drop each into the Flu A and B tubes. RT‐LAMP, followed by LFIA. The LAMP amplicon containing biotin‐dUTP is bound to the streptavidin‐conjugated gold on the conjugate pad and captured by immobilized avidin on the T‐line. Biotin‐BSA is loaded on the C‐line.	33–38 min, including sample preparation for 3 min, 25 min for RT‐LAMP at 58°C, LFIA assay for 5–10 min	IAV RNA (1 × 10^5^ copies) and IBV (1 × 10^6^ copies)	—	[33]
ASFV	CRISPR/Cas12a‐LFIA system. Specific crRNAs target the ASFV *B646L* gene. CRISPR/Cas12a cleaves dsDNA and triggers robust, nonspecific trans‐cleavage of ssDNA. The ssDNA reporter was labeled with digoxin and biotin at the 5^′^ and 3^′^ termini, respectively. Mouse anti‐digoxin antibody‐conjugated GNPs are loaded on the conjugate pad. Streptavidin and rabbit anti‐mouse IgG were used for the C‐ and T‐lines, respectively.	36–56 min, including 3 min for isolating DNA, 20 min for RAA, 30 min for CRISPR/Cas12a reaction, and 3 min with LFIA.	The LOD of this CRISPR/Cas12a‐LFIA was ~32 copies of ASFV DNA and could be improved to 20 copies with RAA.	100% specificity and sensitivity compared with qPCR.	[34]
	GNP‐anti‐p30 mAb on the conjugate pad, anti‐p30 mAb on the T‐line, and anti‐mouse IgG on the C‐line.	15 min	Required the addition of 3% H_2_O_2_ in the sample to limit matrix interference and prevent false positive results.	100% specificity and 93% sensitivity compared with qPCR for samples with high viral load (Ct < 27). Stable 7 days at 37°C and 3 months at room temperature.	[35]
CPV	For colloidal‐gold‐based LFIA: colloidal‐gold‐mAb on the conjugated pad. For TRF‐based LFIA: fluorescence‐mAb and rabbit IgG on the conjugated pad. Goat anti‐mouse antibodies and viral‐specific mAb 6A8 were sprayed on the C and the T line, respectively.	10–15 min	For colloidal gold‐based and TRF‐based LFIA, positive results for CPV (TCID_50_ = 10^3.9^/0.1mL) are achieved at dilutions of 1:16 and 1:160, respectively.	Specificity: 100%. The correlation rate was 100% and 95% for HA and qPCR, respectively. Stable for 4–5 months at RT and up to 6–7 months at 4°C.	[36]
	Competitive LFIA: colloidal gold‐labeled recombinant capsid protein of CPV‐2 and rabbit IgG on the conjugate pad, mouse anti‐CPV IgG on the T‐line, mouse anti‐rabbit IgG on the C‐line.	10 min	Antibody as low as 375 ng/mL of CPV antibody, which equals a 1:40 HAI titer	Specificity: 100%	[37]
SFTSV	The recombinant nucleoprotein of SFTSV is fused with magnetic beads, enabling the separation of virus‐specific antibodies from various animal samples via magnetic separation. Then, the isolated antibody–antigen complex was applied to the LFIA strip, where the T‐line was coated with protein A/G and the C‐line with antinucleoprotein antibodies.	15 min	Antibody as low as 0.5 OD value of ELISA	Can detect anti‐N antibodies in mouse, rabbit, and monkey sera with high sensitivity (100%) and specificity (84.2%) compared with ELISA.	[38]
	Catalytic hairpin assembly (CHA) based LFIA: Two probes (H1 and H2) are hairpin‐structured. H1 is capable of hybridizing to both viral RNA and H2 via distinct sequences. The 5^′^ ends of H1 and H2 are conjugated with biotin and digoxigenin, respectively. When target RNA is present in the sample, H1 forms a complex with the target (H1‐Target complex), which then binds to H2 to generate an H1‐H2 complex. This complex can interact with streptavidin and fluorophore‐labeled NPs preloaded on the conjugate pad. Finally, the complex is captured and detected by anti‐digoxin antibodies on the T line.	30 min	500 copies/mL	Specificity: 100%. Consistent with the results of RT‐PCR.	[39]
MPXV	Fe_3_O_4_‐GNPs are conjugated with mAb specific to MPXV for virus enrichment via magnetic separation. The enriched complex was then added to the LFIA, which featured a specific MPXV mAb immobilized on its T‐line and goat anti‐human IgG on the C‐line.	15 min	5 ng/mL	Specificity: 100%. The GNPs provide dual‐mode colorimetry and photothermal quantification.	[40]
NiV	Digoxin and FAM are labeled on the 3^′^ or 5^′^ end of the primer, respectively, for one‐step RT‐PCR. For LFIA, colloidal gold‐anti‐FAM antibody, anti‐digoxin antibody, and sheep anti‐mouse polyclonal antibody are on the conjugate pad, T‐line, and C‐line, respectively.	58 min, including 10 min for nucleic acid extraction, 47 min for RT‐PCR, and 1 min for LFIA	199.1 copies of viral nucleic acids	Specificity: 100%. Consistent with the results of RT‐PCR.	[41]
SVA	The conjugate pad is loaded with gold‐conjugated SVA 3AB protein and gold‐conjugated mouse IgG. The T‐line is loaded with goat anti‐pig IgG, and the C‐line is sprayed with anti‐mouse IgG.	15 min	2log_2_ of SVA‐specific IgG	Specificity: 100%. 97.97% sensitivity and 90.00% specificity compared to the viral neutralization assay. A sensitivity of 98.62% and a specificity of 86.79% compared to the ELISA.	[42]
MERS‐CoV	The cellulose nanobeads (CNB)‐LFIA: CNB conjugated detection antibody was on the conjugate pad. The capture antibody and anti‐mouse IgG antibody were on the T‐ or C‐line. TRF‐LFIA: The biotinylated capture antibody is on the biotin pad, Europium chelate particle‐labeled detection antibody is on the conjugated pad, the streptavidin and the control antibody are on the T‐ or C‐line, respectively.	20 min	CNB‐LFIA: 2.5–10 ng/mL for different capture mAb pairs. TRF‐LFIA: 0.1–1 ng/mL for different capture mAb pairs; 5 × 10^4^– 5 × 10^5^ copies/mL of MERS‐CoV.	Specificity: 100%. TRF‐LFIA exhibits a 25‐fold sensitivity increase relative to the CNB‐LFIA.	[43]
PCV2	The colloidal gold‐pAb was on the conjugate pad, and pAb was on the T‐line. Goat anti‐rabbit mAb is on the C‐line.	10–15 min	5 ng/mL of recombinant viral protein	Specificity: 100%. The positive rate was 88.9% compared with the commercial ELISA Kit. 4°C for 24 w	[44].
FCoV	The GNP‐conjugated mAb was applied to the conjugate pad, and another mAb was applied to the T‐line. The C‐line is labeled with goat anti‐mouse IgG. The mAbs are specific against the FIPV N protein.	15 min	209.8 ng/mL of recombinant viral N protein	Specificity: 100%. The positive concordance rate of this strip is 70% of that of conventional PCR.	[45]

### 3.1. Detection of Pathogen

**Figure 2:**
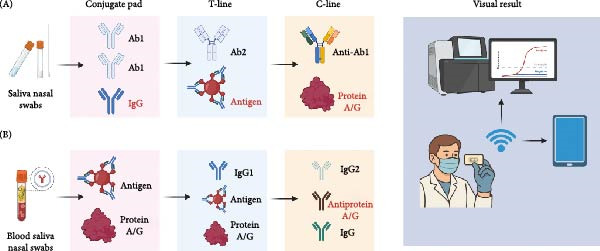
Examples of the LFIA targeting viral antigen (A) and antibody (B). (A) LFIA for antigen detection. Samples are collected using saliva/nasal swabs and dissolved in the liquid in the tube. Then, the sample solution is loaded onto the conjugate pad, and viral antigen (such as virion, virus‐like particle, or viral protein) can be labeled by the reporter‐labeled detection antibody (Ab1) or IgG. After that, the antigen–reporter complex is captured by an immobilized capture antibody (Ab2) or antigen at the T‐line. The C‐line captures excess conjugate via anti‐Ab1 or Protein A/G. (B) Antibody LFIA. Blood, saliva, or nasal samples containing virus‐specific IgG bind reporter‐labeled viral antigen or Protein A/G on the conjugate pad. Immune complexes are retained at the T‐line by immobilized IgG, antigen, or protein A/G. IgG2, anti‐protein A/G, or IgG on the C‐line is responsible for capturing residual conjugate. Both formats can use biotin/streptavidin, fluorescent dyes, gold nanoparticles, and quantum dots (QDs) to label the detection antibody or antigen on the conjugate pad, allowing for visual reading or detection by an instrument or smartphone. Ab/IgG, virus‐specific antibody/IgG; Protein G/A, Staphylococcal protein G/A.

For the detection of viral antigen and nucleic acid, which can be classified as pathogen detection, LFIA based on the sandwich assay is widely used (Figure 2A). This approach is ideal for identifying acute infections, as viral nucleic acids and antigens are present in body fluids throughout the entire course of infection, even detectable in the early stages, which typically precede the full onset of symptoms [21]. For instance, SARS‐CoV‐2 antigen LFIA tests can detect the viral nucleocapsid or spike proteins within 1–3 days of infection [24]. Similarly, Li et al. [32] developed an LFIA that can simultaneously differentiate SARS‐CoV‐2, adenovirus (ADV), and influenza A virus (IAV) in 15–20 min. Chen et al. [41] validated a novel LFIA for on‐site identification of Nipah virus (NiV) nucleic acids, which can detect as low as 199.1 copies of viral nucleic acids. Furthermore, Zhang et al. [36] developed an LFIA strip using a specific mAb against Canine parvovirus (CPV) for virus detection, which can complete the detection within 15 min with a similar positive/negative rate to that of qPCR.

Beyond its application in detecting human and animal viruses, LFIA also serves as a viable tool for identifying plant viruses. As reported by Greeshma et al. [46], the LFIA can detect piper yellow mottle virus and cucumber mosaic virus in peppers with a limit of detection (LOD) of 100 ng and 50 ng, respectively. Selvarajan et al. [47] developed an LFIA for Banana bract mosaic virus detection, which can finish the detection within 5–10 min with a LOD of about 10 ng and high sensitivity (99.04%) and specificity (100%).

### 3.2. Detection of Viral Antibody

LFIA for antibody detection is designed to evaluate the host’s immune response against viral infection by targeting virus‐specific IgM or IgG [21, 25, 48, 49]. Generally, LFIA captures these antibodies using immobilized viral antigens on the T‐line (Figure 2B). In this circumstance, IgM detection helps identify recent infections, while IgG detection indicates postexposure immunity or prior exposure. Therefore, the LFIA targeting antibody investigation is particularly valuable in serological surveys. Unlike antigen tests, antibody‐targeting LFIA often uses blood or serum samples because antibodies are more concentrated in the blood. The assay design may also include blocking reagents to reduce cross‐reactivity with other viral antibodies. Notably, a high proportion of virus‐specific IgG in the blood sample can interfere with the detection of IgM using an LFIA. As reported by Salmanli et al. [37], a competitive LFIA using colloidal gold‐labeled recombinant capsid protein of CPV‐2 can detect as low as 375 ng/mL of CPV antibody, which equals a 1:40 hemagglutination inhibition (HAI) titer. Another group developed an LFIA strip to detect Equine infectious anemia virus (EIAV) antibodies using a fusion protein, p26‐gp90, as the label antigen on the conjugate pad and the detection antigen on the T‐line [50]. This strip has a 91.41% positive concordance rate compared to the competitive ELISA (cELISA).

Together, antigen‐targeted LFIA enables rapid outbreak response by identifying active infections, while antibody LFIA supports long‐term health planning through immunity monitoring. This flexibility, combined with LFIA’s inherent portability and low cost, makes it an important tool in both acute viral disease management and surveillance.

### 3.3. LFIA Application in Human, Animal, and Zoonotic Viruses

LFIA for viral detection is distinct based on its target hosts, such as humans, animals, or zoonotic hosts, with design optimizations for sample types, detection objectives, and end‐user needs. For human viruses, LFIA prefers clinical utility for POCT, focusing on viruses that primarily circulate in human populations and optimizing for noninvasive human samples, such as nasal and oropharyngeal swabs. A typical example is the QuickVue Influenza A + B LFIA Kit (Quidel Corporation, USA), widely used in primary care to detect antigens of IAV and influenza B virus (IBV) from nasal swabs [51]. The kit delivers qualitative results within 10 min, enabling clinicians to promptly prescribe antivirals, which are critical for reducing severe outcomes in high‐risk patients. Another instance is the LFIA for SARS‐CoV‐2 antigen detection, such as the Abbott Panbio COVID‐19 Ag Rapid Test Device [52]. These tools were pivotal during the pandemic for mass screening, allowing rapid isolation of infectious individuals to control human‐to‐human transmission.

Animal‐targeted viral LFIAs emphasize field adaptability for the surveillance of livestock or poultry viral diseases (including both viral antigens and antibodies), accommodating harsh conditions and animal‐specific sampling. As such, samples such as animal feces, oropharyngeal swabs, blood, milk, or even tissues from diseased animals can be used for LFIA detection. The strips are resistant to environmental variability (such as temperature changes) and require no laboratory equipment, making them ideal for on‐site screening in remote farms to support rapid culling decisions during epizootics. For example, an LFIA based on nonstructural protein 3AB of Senecavirus A (SVA) shows comparable performance to the virus‐neutralizing assay and an in‐house ELISA in detecting viral antibodies in serum samples [42]. Additionally, feline immunodeficiency virus (FIV) antibody test kits can distinguish natural FIV infection between antibodies from vaccinated and naturally infected cats with high sensitivity (100%) and specificity (98%–100%) [53, 54].

Zoonotic viral LFIAs bridge animal reservoir monitoring and human infection prevention, targeting viruses that transmit between animals and humans. Therefore, both viral antigen and antibody surveillance are crucial for controlling and preventing zoonotic diseases. For example, the Goldsite Monkeypox Virus (MPXV) Antibody IgG/IgM Kit, developed by Goldsite Diagnostics Inc., can rapidly determine the history of MPXV infection within 15 min (https://en.goldsite.com.cn/), which can provide an emergent evaluation of individuals from the epidemic regions. Notably, a key feature of zoonotic viral LFIA is its ability to simultaneously detect samples from a variety of different animal species and/or even human samples. For example, Chang et al. [38] established an LFIA for the detection of severe fever with thrombocytopenia syndrome virus (SFTSV), a zoonotic virus, by targeting viral antibodies using recombinant viral nucleoprotein (rNP). This strip can detect anti‐NP antibodies in sera from rabbits, mice, and monkeys.

Therefore, host‐specific design and applications of LFIA make it a versatile tool for detecting viruses across human, animal, and zoonotic diseases.

## 4. Advantages and Limitations of LFIA in Viral Disease Diagnosis

### 4.1. Advantages of LFIA

Several advantages enable LFIA to be used in the diagnosis of viral diseases and serological surveillance (Table 2) [22, 55–57]. First, LFIA requires minimal sample preparation and can be performed by nonexperts with little or no training. The test results can be visually interpreted, eliminating the need for a complex instrument and a technical expert. Second, most LFIA tests can provide results within 15–30 min, which is much faster than traditional laboratory‐based methods, such as RT‐PCR or ELISA. This rapid performance is crucial for the timely detection and control of viral diseases, especially in emergency or outbreak situations.

**Table 2:** Overall summary of advantages, disadvantages, and solutions of LFIA.

Advantages	Disadvantages	Solutions
1. Simplicity and ease of use: Requires minimal sample pretreatment and no specialized technical training, with visualized results.	1. Suboptimal sensitivity: Generally lower detection limits than RT‐PCR, risking false negatives for low viral load samples.	1. Signal amplification: Use GNPs, QDs, or lanthanide fluorophores to enhance signal intensity; nanomaterials designed to bind to different epitopes of target viruses or their proteins to boost target binding affinity; Combine nucleic acid amplification with LFIA.
2. Rapid: Delivers results within 10–30 min.	2. Limited multiplexing: Traditional strips only detect 1–2 analytes, unable to distinguish multiple viral strains or coinfections simultaneously.	2. Multiplexing strategies: Use multiple color/fluorescent‐labeled probes for multitarget detection; integrate SERS, microfluidics, or multichannel strips to expand analyte capacity.
3. Cost‐efficiency: Low per‐test costs and no dependency on expensive instrumentation, suitable for large‐scale screening.	3. Reagent dependency: Performance varies with antibody/antigen quality; batch‐to‐batch inconsistencies affect reliability; unstable storage in the field.	3. Instrument‐assisted readout: Apply handheld optical readers for quantitative signal measurement; connect to AI‐assisted image processing, IT‐linked POC devices, mobile/cloud data integration platforms for remote data analysis.
4. Portability: Compact, lightweight strip design; no refrigeration needed, enabling on‐site detection in remote clinics and field settings, etc.	4. Subjective readout: Visual interpretation of weak signals leads to interobserver variability.	4. Sample enrichment and sample pretreatment: Use filtration/centrifugation/magnetic separation to remove contaminants from the animal samples; optimize buffer systems or add blocking agents to reduce matrix interference; use freeze‐dried reagents. Proper medical waste management of LFIA strips, including chemical disinfection and autoclaving. A biodegradable strip combined with eco‐friendly reagents.
5. High specificity: Well‐designed assays exhibit strong target selectivity (e.g., via specific antibody/antigen probes), minimizing false positives.	5. Matrix interference: Sample contaminants, such as animal fecal swabs, cause nonspecific binding or flow disruption, distorting results.	

Furthermore, the cost of the LFIA strip is lower than that of other diagnostic techniques, as it eliminates the need for expensive laboratory equipment or highly trained personnel, making it an attractive option for resource‐limited situations and antigen/antibody screening programs [58]. Moreover, LFIA strips are small, portable, and can be stored at room temperature. They can be easily transported and used in various conditions, including remote areas, clinics, and even at home, enabling on‐site POCT. Additionally, when properly designed, LFIA can exhibit high specificity for the target virus, thereby minimizing false‐positive results. The use of specific antibodies or nucleic acid probes in the assay ensures that only the target virus is detected, reducing the interference from other substances in the sample. As reported by Nicol et al. [59], the specificity of LFIA for IgG detection is greater than that of ELISA.

These advantages make LFIA a versatile and accessible tool for on‐site management of viral diseases across various settings.

### 4.2. Limitations of LFIA

There are limitations for LFIA that need to be addressed to enhance its specificity and sensitivity [22, 57, 60]. Although LFIA has made significant progress in sensitivity, it still generally has lower sensitivity compared to reference methods, such as qPCR and ELISA, which may lead to false‐negative results at low levels of infection, particularly in the early stages of the infection [20, 44, 45, 50, 61, 62]. For example, Ding et al. [44] established an LFIA for porcine circovirus 2 (PCV2) detection using colloidal gold‐labeled polyclonal antibodies (colloidal Au‐pAb) as the probe and the pAb as the capture antibody. This assay exhibits an 81% concordance rate with that of the commercial ELISA. Similarly, another group reported an LFIA for Feline coronavirus (FCoV) detection based on the mAb against the N protein of feline infectious peritonitis virus (FIPV) [45]. However, the positive concordance rate of this strip is only 70% of that of qPCR. Vu et al. [63] compared the sensitivity and specificity of portable qPCR and LFIA for ASF virus (ASFV) detection. They found that the portable qPCR showed similar performance to the standard qPCR in detecting the virus from blood and oral swabs. However, the LFIA exhibited 100% specificity but a reduced sensitivity (65.9%) compared with the standard qPCR. Additionally, many LFIA are visually interpreted. However, when the signal is weak, this may lead to inconsistent results and potential misdiagnosis [21, 58]. Thus, enhancing LFIA sensitivity is crucial for rapid disease assessment.

Furthermore, in the field, coinfections with diverse pathogens are common in the animal [30, 64]. For example, PCV2 is usually coinfected with other pathogens, such as PCV3, porcine reproductive and respiratory syndrome virus (PRRSV), porcine parvovirus, pseudorabies virus (PRV), and/or swine influenza virus, resulting in more serious diseases in pigs [64]. Notably, several viruses, including respiratory viruses, gastrointestinal viruses, and blood‐borne viruses, can result in similar clinical signs and symptoms, which must be differentiated for effective prevention and control [61]. However, most traditional LFIA strips can only detect one or two analytes, which limits their ability to simultaneously detect multiple viral infections or distinguish between different types of the same virus.

Moreover, the performance of LFIA highly depends on the quality of the antibodies, antigens, and other reagents used in the assay. Variations in reagent quality can affect the sensitivity and specificity of the assay.

In addition, the presence of certain substances in the sample, such as lipids, proteins, or other contaminants, may cause nonspecific binding, thereby affecting the migration of the sample through the strip or interfering with the formation of the specific antigen–antibody complex, which can result in false‐positive or false‐negative results.

These limitations collectively hinder the reliability and field reproducibility of LFIA, underscoring the need for targeted technical improvements.

### 4.3. Solutions for the Limitations of LFIA

#### 4.3.1. Signal Amplification

Nanomaterials, including GNPs, QDs, nanobodies (NBs), and MNPs, significantly enhance the sensitivity of LFIA due to their small size and high stability, thereby improving target binding and signal‐to‐noise ratios [25, 38, 42, 65]. For example, Jeong et al. [25] constructed an LFIA strip by conjugating SARS‐CoV‐2‐specific NBs to GNPs and found that this strip showed 25‐fold higher sensitivity than the commercial antibody‐based LFIA kit. Moreover, lanthanide fluorophores, including europium, terbium, and samarium, can emit fluorescence with a long decay time [43], making them suitable for sensitive detection with increased signal‐to‐noise ratios. The time‐resolved fluorescence (TRF)‐based LFIA strip for Middle East respiratory syndrome (MERS) is characterized by using a biotin‐labeled capture antibody loaded on the biotin pad, followed by an europium‐fused detection antibody on the conjugation pad, and streptavidin on the T‐line [43]. When the viral analyte is added to the sample pad, the virus can be captured by the biotin‐labeled capture antibody, forming a sandwich structure with the Europium‐fused detection antibody, and immobilized by the streptavidin on the T‐line. This strip showed a 25‐fold higher sensitivity compared to conventional LFIA.

Another strategy to enhance the sensitivity of LFIA involves the use of nanomaterials designed to bind to different epitopes of target viruses or their proteins. For example, Li et al. [66] construct a multiple aptamer‐based QD LFIA, which can simultaneously target different sites of the virus protein with multiple high‐affinity aptamers to enhance the sensitivity. The assay can achieve an LOD of 1.427 pg/mL for viral nucleocapsid and 1643 U/mL for the virions. The triple‐antibody sandwich LFIA reported by Hu et al. [26] showed that the LOD of the assay targeting the nucleocapsid or receptor‐binding domain (RBD) of SARS‐CoV‐2 is 12.01 and 6.51 pg/mL.

Other technologies, such as plasmonic scattering, also hold promise for enhancing the LFIA sensitivity [67, 68]. As reported by Lee et al. [67], a transparent membrane paired with a light‐absorbing layer positioned beneath it was integrated into LFIA to reduce reflection of incident light. This design achieved a signal enhancement 2600–4400 times greater than that of conventional LFIA.

**Figure 3:**
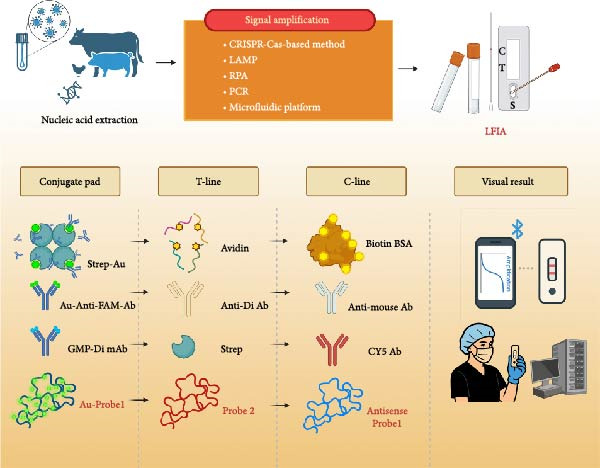
Examples of the LFIA targeting viral nucleic acids integrated with signal amplification. Nucleic acids from clinical/animal samples are extracted and amplified via LAMP, RPA, or PCR, or modified by CRISPR‐Cas with or without the use of microfluidic platforms, generating labeled products that can be detected using an LFIA strip. (1) Biotin‐labeled nucleic acids bind to the Strep‐Au on the conjugate pad and are captured by the avidin on the T‐line. Biotin‐BSA on the C‐line is used for binding with excess Strep‐Au. (2) FAM‐ and Di‐dual labeled nucleic acids are reacted with Au‐anti‐FAM Ab on the conjugate pad and captured by the anti‐Di Ab on the T‐line, resulting in an Au‐anti‐FAM antibody‐viral amplicon‐anti‐Digoxin antibody structure. Anti‐mouse Ab on the C‐line is used for binding Au‐anti‐FAM Ab. (3) LAMP is performed using virus‐specific primers Biotin‐FIP and Digoxin‐BIP, while the primers for the reference gene were CY5‐FIP and Digoxin‐BIP. Then, the amplicons can be fused with GMP‐Di mAb on the conjugated pad. Subsequently, the biotin‐labeled amplicons were immobilized by streptavidin on the T‐line, and the CY5‐labeled amplicons bound to the CY5 antibody on the C‐line. (4) The target nucleic acids are first amplified via LAMP, and the resulting amplicons are then loaded on the strip. The reporter probe (Au‐probe1) hybridizes with the target sequence, while the capture probe (probe2) specifically recognizes the single‐stranded loop regions of LAMP amplicons. This dual‐recognition event drives the formation of a hybridization complex, which accumulates to generate visible signals. Results can be interpreted either visually or quantitatively via dedicated instruments or smartphone‐based detection systems. Ab, antibody; Au, gold nanoparticle; BSA, bovine serum albumin; Cy5, cyanine‐5; Di, digoxin; FAM, 6‐carboxyfluorescein; GMP, gold magnetic particle; mAb, monoclonal antibody; Strep, streptavidin.

To improve the sensitivity and specificity of the viral nucleic acid‐based LFIA, multiple groups contribute to combining the LFIA with nucleic acid amplification techniques (such as isothermal amplifications and PCR) and/or Clustered Regularly Interspaced Short Palindromic Repeats (CRISPR)‐Cas system (Figure 3) [29, 33, 34, 41]. Notably, combining isothermal amplifications, such as loop‐mediated isothermal amplification (LAMP), recombinase‐aided amplification (RAA), and recombinase polymerase amplification (RPA), with LFIA enhances its sensitivity while retaining the portability and ease of operation, eliminating the requirement for specialized instruments [33, 35, 69]. For example, Jang et al. [33] developed an RT‐LAMP‐LFIA strip to detect the influenza virus. In this system, the amplicons can be labeled with biotin‐dUTP during amplification and captured by streptavidin‐GNPs on the conjugate pad, followed by immobilization with avidin on the T‐line. Chen et al. [29] reported the use of LFIA for PED virus (PEDV) detection by labeling‐specific primers with digoxin and FAM, which generated digoxin‐ and FAM‐conjugated amplicons after a one‐step RT‐PCR. Thereafter, the amplicon can be captured by the colloidal gold‐anti‐FAM antibody on the conjugated pad and immobilized by the anti‐digoxin antibody, resulting in a colloidal gold‐anti‐FAM antibody‐viral amplicon‐anti‐digoxin antibody structure on the T‐line. Similarly, Fan et al. [70] developed a method for detecting IAV and IBV by combining RT‐LAMP, LFIA, and gold MNPs. In this system, the LAMP primers targeting IAV and IBV were fused with biotin (Biotin‐FIP) and digoxin (Digoxin‐BIP), while the primers for the reference gene were labeled with digoxin (Digoxin‐BIP) and CY5 (CY5‐FIP). Then, the amplicons can be fused with gold magnetic particles‐conjugated digoxin antibodies on the conjugated pad. Subsequently, the biotin‐labeled amplicons were immobilized by streptavidin on the T‐line and the CY5 antibody on the C‐line. Clinically, this RT‐LAMP‐LFIA displayed complete consistency (100%) with RT‐qPCR results. Reo et al. [71] developed a hybridization‐specific LFIA, which employs thermal lysis to extract DNA, performs direct LAMP, and uses an LFIA strip preloaded with capture probes and reporter probes linked to GNPs. The capture probe (probe2) specifically recognizes the single‐stranded loop regions of LAMP amplicons, and the reporter probe (probe1) produces colorimetric signals, facilitating sensitive on‐site identification. Estrela et al. [27] described a multiplex RT‐LAMP‐LFA assay for differentiating SARS‐CoV‐2 variants, particularly the Omicron BA.1 and BA.2 lineages. This assay is characterized by its fast and cost‐effectiveness, alongside high sensitivity, specificity, and accuracy in detecting both BA.1 and BA.2. Moreover, Prescott et al. [69] constructed an RT‐RPA‐LFA platform that enabled the sensitive and timely detection of DENV2 from serum samples. Besides, RT‐LAMP combined with CRISPR/Cas12a and LFIA enables effective and sensitive detection of transmissible gastroenteritis virus (TGEV) RNA at a concentration of 10^2^ copies/µL [72]. Notably, in some methods, it is necessary to pre‐extract viral nucleic acids and perform target gene amplification before detecting them with LFIA. In contrast, newly developed strategies have introduced the “one‐pot” method, wherein viral nucleic acid extraction and target gene amplification are conducted in a single tube. This design not only eliminates the risk of contamination but also shortens the detection time. Besides, for wildlife samples, such as bat oral swabs and rodent tissue, LFIA with preamplification (such as RT‐LAMP‐LFIA) can enhance sensitivity for low‐abundance viruses, enabling on‐site detection of potential zoonotic pathogens, such as coronaviruses.

To improve the specificity, dual or multiple recognition elements are used in the LFIA. For example, Le et al. [73] developed an LFIA by adding biotinylated nucleic acid aptamers and a GNPs–antibody complex to the conjugated pad, which can simultaneously bind to a specific virus and be captured by streptavidin on the T‐line. Therefore, the LFIA can discriminate between different virus subtypes based on the aptamer and antibody preloaded on the conjugated pad. Chen et al. [39] demonstrated that two hairpin probes targeting SFTSV can boost the detection sensitivity of LFIA for this virus. In this detection system, the two probes (designated H1 and H2) are hairpin‐structured. Specifically, H1 is capable of hybridizing to both viral RNA and H2 via distinct sequences. The 5^′^ ends of H1 and H2 are conjugated with biotin and digoxigenin, respectively. When target RNA is present in the sample, H1 first forms a complex with the target (H1‐Target complex), which then binds to H2 to generate an H1–H2 complex. This resulting complex can interact with streptavidin and fluorophore‐labeled nanoparticles preloaded on the conjugate pad. Finally, the complex is captured and detected by anti‐digoxin antibodies on the T line.

Additionally, NBs and affibodies, valued for small size and stability, also replace capture/detection antibodies in antigen‐targeted LFIAs [68, 74]. As reported by Sadler et al. [68], GNPs were conjugated with biotinylated affibodies, serving as the agent for antigen binding. Meanwhile, a second biotinylated affibody was immobilized on the T‐line to act as the capture probe, and the C‐line was preloaded with streptavidin.

These results demonstrate that the sensitivity and specificity of LFIA are enhanced through the use of nanomaterials, amplification technologies, and multirecognition strategies, thereby strengthening its on‐site detection capabilities.

#### 4.3.2. Multiplex Detection Strategies

LFIA for multiplex detection of animal viral coinfections, differentiating between various viruses in the same specimen, and distinguishing viral diseases that present with similar symptoms is crucial in ensuring accurate disease diagnosis.

To overcome the limited multiplexing capability of LFIA, several strategies have been developed. One approach is to use different‐colored or fluorescently labeled probes for each target analyte, allowing for the detection of multiple viruses or different virus subtypes on a single strip simultaneously [75–78]. For example, Wu et al. [30] developed a multiplexed fluorescence LFIA for differentiating PEDV, porcine deltacoronavirus (PDCoV), and TGEV. The assay is characterized by preloaded europium nanoparticles‐conjugated antibodies specific to PDCoV, TGEV, and PEDV on the conjugated pad, and virus‐specific antibodies on three T‐lines. Li et al. [32] reported a multiplex fluorescence LFIA for simultaneous differentiation of ADV, SARS‐CoV‐2, and IAV using QD nanobeads‐conjugated antibody as a detection probe, which can form an antigen–antibody complex with the target and be captured by specific capture antibodies on the T‐line. Compared to the colloidal gold LFIA targeting these viruses, the sensitivity of this assay is 200, 417, and 1220 times higher, respectively. Similarly, Ye et al. [8] integrated reverse transcription RAA (RT‐RAA) with LFIA to differentiate PEDV, PDCoV, and TGEV. A key feature of this system is that reverse primers targeting the three viruses are labeled with distinct fluorophores (Alexa Fluor 488, digoxigenin, and 6‐carboxyfluorescein), while specific probes bear a biotin label. The RAA reaction is completed within 10 min, after which the amplicons are mixed with streptavidin‐coated colloidal gold. This mixture is then added to a test strip where the T line is immobilized with antibodies corresponding to each fluorophore, enabling visual differentiation of the three viruses. As expected, this method achieves 100%, 99.1%, and 99.1% consistency with qPCR in clinical testing, respectively, suggesting that multiplex detection or differentiation using LFIA is feasible in the field.

Another strategy is to combine LFIA with microfluidic technology or surface‐enhanced Raman spectroscopy (SERS), which enables the integration of multiple assays on a single chip, thereby further enhancing the ability for multiple diagnoses [24, 79]. For example, a power‐free microfluidic device was constructed for the timely detection of HBV, featuring nucleic acid extraction based on magnetic bead‐immiscible filtration assisted by surface tension, LAMP amplification, and LFIA detection [70]. This device achieves an LOD of 3.04 copies/μL, with 100% sensitivity and specificity. Additionally, multichannel or multistrip LFIA devices, where each channel or strip is dedicated to detecting a specific analyte, are also designed [80, 81].

Therefore, diverse strategies, such as labeled probes, microfluidic integration, and multichannel designs, can enhance the multiplexing ability of LFIA for accurate detection of animal viruses.

#### 4.3.3. Instrument‐Assisted Readout

To reduce the subjectivity in visual interpretation, instrument‐assisted readout methods can be used [82–84]. Handheld readers integrated with optical sensors, artificial intelligence (AI)‐assisted image processing, and IT‐linked POC devices can accurately measure the signal intensity on the LFIA strip [85–87]. These instruments can provide quantitative outcomes with minimized human‐induced errors. More advanced readers can also be connected to a mobile device or cloud‐based data systems, enabling remote data transfer and analysis. For example, Liu et al. [83] have developed an automated detection method for the IAV H1N1, which combines nanozyme LFIA with a centrifugal microfluidic chip and optoelectronic sensing. This detection can be finished within 16 min (LOD = 50 pg/mL), exhibiting a 2.5‐fold improvement over that of the naked‐eye LFIA. Tong et al. [85] developed an AI‐assisted colorimetric LFIA for sensitive, quantitative detection of COVID‐19 neutralizing antibodies (nAbs). This system overcomes the subjectivity of naked‐eye reading by employing image analysis algorithms to quantify the color intensity of test strips, enabling precise measurement of nAb titers. It demonstrates high sensitivity and specificity, matching the performance of commercial gold‐standard methods, and thus reliably distinguishes seropositive from seronegative samples. With rapid results and ease of use, the platform is well‐suited for POCT, large‐scale serological surveillance, and postvaccination immunity evaluation. Moreover, Behrouzi et al. [86] introduce a plasmonic coffee‐ring biosensing platform integrated with AI for POC diagnostics. This system is based on the plasmonic coffee‐ring effect, where metallic nanoparticles form ring‐shaped assemblies during sample drying, thereby amplifying biosignals and enhancing sensitivity for target analytes. Then, AI algorithms analyze these plasmonic patterns, enabling the quantitative and high‐precision detection of SARS‐CoV‐2 nucleocapsid within 12 min while eliminating subjective human judgment. It features rapid sample processing, portability, and ease of use, matching laboratory‐based methods in detecting the virus in POCT. The design also supports scalable multiplex detection, facilitating broad applications in disease screening, serological surveillance, and on‐site monitoring.

SERS‐based LFIA, as well as AI‐aid platforms, further enhance its sensitivity, enabling readout via naked eye or handheld readers [31, 87–90]. Shi et al. [31] constructed an SERS LFIA based on the gold nanoantennas and Raman molecule 4‐MBA. In this strip, the 4‐MBA and PEDV antibodies were immobilized on the surface of the gold nanoantennas and preloaded on the conjugated pad to bind PEDV virions. Then, the PEDV antibody on the T‐line detects the virus–nanoparticle complex, and the LFIA can be examined via naked eye or a Raman spectrometer, with LOD of about 1.0 × 10^2^ and 1.0 TCID_50_/mL, respectively. Additionally, 4‐MBA and Au@Ag nanoparticle‐based LFIA have also been reported for the detection of PEDV and porcine group rotavirus (PoRV) [91, 92]. Notably, digital SERS‐based LFIA can detect as low as 0.001–10 pg/mL viral protein in the nasal swabs, with the LOD of about 0.9 fg/mL [93]. In an LFIA strip constructed by Li et al. [94], mAbs targeting IAV and IBV were labeled with distinct types of Raman nanospheres to capture the respective viruses in the sample. A mixture of antibodies against both viruses was immobilized on a single T‐line to detect the formed antibody‐antigen complexes. As a result, this LFIA can identify and distinguish between IAV and IBV based on the distinct performance characteristics of the Raman nanospheres used. Recently, Zhao et al. [87] developed an AI‐driven integrated SERS‐LFIA system that advances automated viral diagnostics via SERS image recognition and deep learning. This system exhibits high accuracy, specificity, and reproducibility, enabling the differentiation of viral subtypes or coinfections by identifying unique SERS spectral signatures. Its integrated design balances portability, fast results, and ease of use, rendering it suitable for POCT and large‐scale viral surveillance.

These results indicate that instrument‐assisted and SERS‐based readouts enhance the objectivity and sensitivity of LFIA, thereby advancing the accuracy of viral detection.

#### 4.3.4. Sample Enrichment, Sample Pretreatment, and Waste Management

To minimize interference from the sample matrix, such as fecal or nasal swabs, suitable sample pretreatment methods can be applied. For example, filtration, centrifugation, magnetic separation, or dilution of the sample can be used to remove contaminants or reduce the concentration of interfering substances. For example, Ren et al. [40] conjugated an mAb specific to MPXV onto the surface of Fe_3_O_4_‐GNPs for virus enrichment via magnetic separation. The enriched complex was then added to the LFIA, which featured a specific MPXV mAb immobilized on its T‐line. The results were detectable through both colorimetry and photothermal quantification, with the LOD as low as 5 ng/mL for the visual detection. Chang et al. [38] conjugated the recombinant nucleoprotein of SFTSV to magnetic beads, allowing for the separation of virus‐specific antibodies from various animal samples via magnetic separation before the assay. Then, the isolated antibody–antigen complex was applied to the LFIA strip, where the T‐line was coated with protein A/G and the C‐line with antinucleoprotein antibodies. This LFIA strip can detect antinucleoprotein antibodies in mouse, rabbit, and monkey sera with high sensitivity (100%) and specificity (84.2%). A similar setup was also reported in the LFIA for the simultaneous detection of IAV, SARS‐CoV‐2, and respiratory syncytial virus [28].

Furthermore, Zou et al. [95] inserted a filter pad between the sample pad and conjugated pad in the latex bead‐based LFIA to minimize the interference of fecal impurities, which exhibits increased performance compared to that of the colloidal gold and fluorescent LFIA for PEDV diagnosis.

Additionally, reagent stability and batch consistency need to be considered for the LFIA platform. Strategies, including the development of freeze‐dried reagents, specific blocking agents, or buffer systems, can preserve the stability of both reagents and test strips, minimize nonspecific binding, and enhance the performance of LFIA in complex samples [32]. Therefore, sample pretreatment and optimized reagents can effectively mitigate matrix interference, thereby enhancing the reliability of LFIA in complex specimens.

Notably, epidemics or pandemics, such as the COVID‐19 pandemic, have generated large quantities of medical waste, underscoring the importance of proper medical waste management [96]. As LFIA is designed for virus detection or disease assessment, samples may contain live viruses, posing a risk of disease transmission or public health issues if mishandled. Thus, proper waste management of LFIA strips is critical for on‐site or POCT tests. The conventional strategy for waste strip management is incineration after detection. However, this strip disposal method may cause environmental pollution, high operational costs, and safety risks. Therefore, alternative methods, such as chemical disinfection and autoclaving, are proposed to inactivate viruses before regular waste processing. Besides, a biodegradable strip combined with eco‐friendly reagents is also promising for reducing long‐term environmental impact after disinfection.

## 5. Conclusion and Future Perspective

Over the past decade, LFIA has shown remarkable progress in the rapid diagnosis of viral diseases. Its simplicity, rapidity, cost‐effectiveness, and portability make it an ideal on‐site detection method, especially in resource‐limited settings and for mass screening [97]. However, LFIA still faces some limitations, including lower sensitivity, limited multiplexing capability, and dependence on the quality of the reagents. To address these limitations, continuous efforts are necessary, including the use of nanomaterial‐based signal amplification, multiplexing strategies, quality control of reagents, AI and instrument‐assisted readout, and sample pretreatment.

With further technological advancements, LFIA is expected to play an even more crucial role in the early detection and effective control of viral diseases, thereby contributing to the health of both animals and humans. Future research should also focus on further improving the performance of LFIA, expanding its applications to new viral targets, and integrating it with other emerging technologies to achieve more comprehensive and accurate diagnostic solutions. Moreover, handheld LFIA readers integrated with AI or cloud‐based data processing and IT‐linked POC devices can reduce subjective errors in the visual interpretation of weak signals from low‐viral‐load animal samples. LFIA detection data from multiple farms can be integrated into regional veterinary surveillance platforms, enabling real‐time tracking of viral disease outbreaks and supporting data‐driven control strategies. Besides, for transboundary diseases (such as ASF and peste des petits ruminants), LFIA modified with viral genotype‐specific NBs, rather than traditional antibodies, may enhance resistance to sample degradation during long‐distance transport, thereby addressing the monitoring needs of cross‐border livestock trade.

## Ethics Statement

The authors have nothing to report.

## Consent

The authors have nothing to report.

## Disclosure

All authors agreed to publish this manuscript. The funders had no role in the study design, data collection and analysis, publication decision, or manuscript preparation.

## Conflicts of Interest

The authors declare no conflicts of interest.

## Author Contributions

Conceptualization, writing – review and revision, supervision, funding acquisition: Xu Gao. Data curation: Quanyu Ren, Yan Wang, Haoyuan Ma, Jialiang Xie, Jianyou Jin, Rumeng Tian, and Hao Yu. Writing – original draft preparation: Quanyu Ren and Haoyuan Ma. Figure: Quanyu Ren, Yan Wang, and Haoyuan Ma.

## Funding

This work was financially supported by the National Key Research and Development Program (Grant Number 2023YFD1300103), the Science and Technology Development Plan of Jilin Province (Grant Number YDZJ202203CGZH050), and the Scientific Research Project of the Department of Education of Jilin Province (Grant Number JJKH20250419KJ).

## Supporting Information

Additional supporting information can be found online in the Supporting Information section.

## Supporting information


**Supporting Information** Graphical abstract can be found online in the Supporting Information section.

## Data Availability

All data are included in this published article.
