# Supplementary material for: Recent Advances in Lateral Flow Immunoassay for Rapid Diagnosis of Viral Diseases
Source: Transbound Emerg Dis. 2026 Jan 10;2026:5701806. doi: 10.1155/tbed/5701806 (PMC12790179; doi:10.1155/tbed/5701806)
Supplement: Supplementary file 1 — Supporting Information Graphical abstract can be found online in the Supporting Information section. [file TBED-2026-5701806-s001.docx]

**Graphical abstract**

**
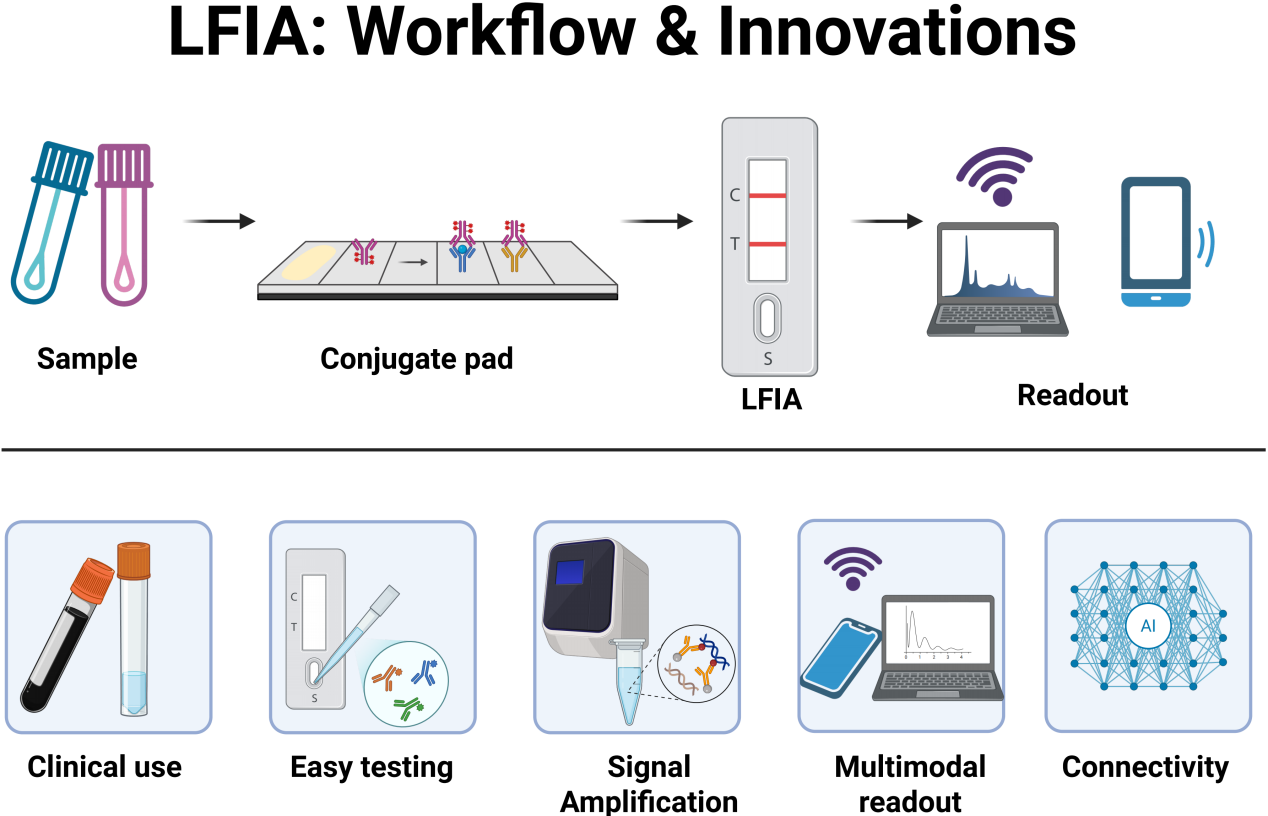
**

Lateral flow immunoassay (LFIA) is a transformative point-of-care diagnostic tool with multiple advantages, including simplicity, rapidity, cost-effectiveness, and portability, particularly in settings with limited healthcare resources. Its ability to detect viral antigens, antibodies, or even nucleic acids made it a versatile tool for both acute infection screening and serological surveillance.
